# Activation of hepatic stem cells compartment during hepatocarcinogenesis in a HBsAg HBV-transgenic mouse model

**DOI:** 10.1038/s41598-018-31406-5

**Published:** 2018-09-03

**Authors:** Beatrice Anfuso, Korri E. El-Khobar, Susan I. Ie, Claudio Avellini, Oriano Radillo, Alan Raseni, Claudio Tiribelli, Caecilia H. C. Sukowati

**Affiliations:** 1 Fondazione Italiana Fegato, AREA Science Park Basovizza, SS14 km 163.5, 34149 Trieste, Italy; 2 Eijkman Institute for Molecular Biology, Jl. Diponegoro 69, 10430 Jakarta, Indonesia; 3 Department of Medical and Biological Sciences, University Hospital, Piazzale Santa Maria della Misericordia 15, 33100 Udine, Italy; 4 Laboratory of Clinical Analysis, Children Hospital Burlo Garofolo IRCCS, Via dell’Istria, 65, 34137 Trieste, Italy

## Abstract

Chronic infection of Hepatitis B Virus (HBV) is one of the highest risk factors of hepatocellular carcinoma (HCC). The accumulation of HBV surface antigen (HBsAg) into hepatocytes induces inflammation and oxidative stress, impairing their replicative ability and allowing the activation of the hepatic stem cells (SC) compartment. This study aimed to understand the involvement of SC during hepatocarcinogenesis in HBsAg-related liver damage, from early injury until HCC. HBsAg-transgenic (TG) and wild type (WT) mouse were followed at several stages of the liver damage: inflammation, early hepatocytes damage, dysplasia, and HCC. Serum transaminases, liver histology, and diagnostic data were collected. The expressions of SC and cancer stem cells (CSC) markers was analyzed by RT-qPCR, immunohistochemistry and Western blot. Starting from 3 months, TG animals showed a progressive liver damage characterized by transaminases increase. The up-regulations of SCs markers Cd34 and Sca-1 started from the beginning of the inflammatory stage while progressive increase of Krt19 and Sox9 and CSCs markers Epcam and Cd133 from early hepatic injury. The expressions of Cd133, Cd34, and Afp were significantly higher in HCC compared to paired non-HCC tissue, in contrast to Epcam and Krt19. Western blot and IHC confirmed the positivity of Cd34 and Cd133 in small cells subpopulation.

## Introduction

Hepatocellular carcinoma (HCC) is the most common primary liver cancer^1^. In around 80% of patients, HCC is preceded by cirrhosis or advanced fibrosis^2^. Overall, 50–55% of HCC cases are attributed to persistent viral infection with Hepatitis B Virus (HBV)^3^. The greater majority of HBV-related HCC develops in cirrhotic liver; however, HBV may act as an oncogenic factor also in its absence^4^. The direct oncogenic effect of HBV is related with the integration of the viral DNA sequence into the host genome; nevertheless, the role of viral proteins in cancer development is still unclear.

The cancer stem cells (CSC) theory postulates that tumor is composed by heterogeneous cells with different grades of differentiation, in which only the CSC are capable of sustaining the tumor and giving rise to proliferating but progressively differentiating cells. The origin of CSC is still uncertain but it was proposed that they may derive from the dedifferentiation of hepatocytes or by mutations occurring in the activated stem cells (SC) niche^4,5^.

The HBV-transgenic mouse C57BL/6J-TG(ALB1HBV)44BRI/J (TG) develops a progressive hepatic damage from chronic hepatitis until HCC as a consequence of the overproduction and the accumulation of the envelope protein (HBV surface antigen, HBsAg) into hepatocytes^6,7^. First damages are detectable at 2–3 months, followed by regenerative phenomena and the appearance of HCC starting from 12 months. Usually, one or two large tumors dominate the process, with numerous smaller tumors scattered throughout^8,9^.

To understand the activation of hepatic SC compartment during liver injury and its role in the process of hepatocarcinogenesis by the insult of HBsAg, we evaluated the expression of different accepted SC markers during the progression of the disease from early hepatic inflammation until HCC.

## Results

### Hepatocarcinogenesis in mouse model

**Figure 1:**
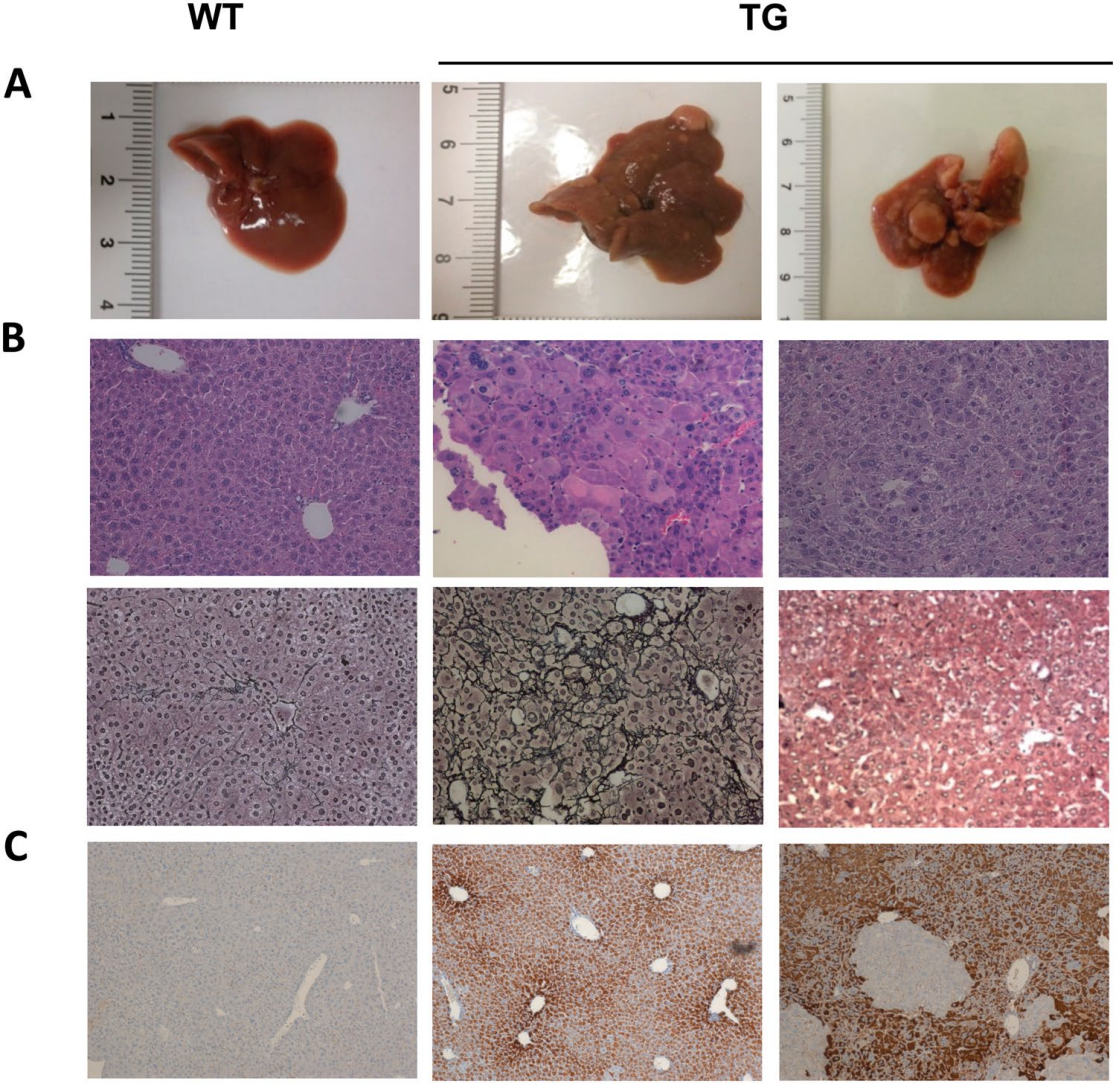
Hepatocarcinogenesis in TG mice. (**A**) Gross appearance of the livers. WT liver is representative of all the ages analyzed. TG animals at 12 and ≥15 months. (**B**) Liver histology. Moving from left to right: representative pictures of H&E (objective 20X) and Gomori (objective 20X) stainings of a WT liver, TG liver with dysplasia, and a well-differentiated HCC. (**C**) HBsAg staining in WT and in TG (objective 10X).

A total of 475 hepatic tissue samples from 107 TG and 97 WT (wild type control) were analyzed at 3, 6, 9, 12, and ≥15 month of age. For each TG mice, at least three different samples from different hepatic lobules were collected. Macroscopically, normal WT livers showed no macroscopic alterations, while TG livers showed a progressive changing both in colour and texture, starting from 3 months of age as previously described^8^. At 12 months, first macroscopic lesions appeared: 20 out of 28 mice (71%) showed lesion of different sizes either as single or multiple nodules. At ≥15 months, the incidence and the size of the tumoral masses became more evident: 19 out of 21 livers (91%) had a visible lesion. Most of the big tumors were vascularised and firm in texture with varied color from white-pale yellow to light brown (Fig. 1A).

In TG mice ≥12 months old, the appearance of ground glass hepatocytes, clear cells, ballooning and necrosis characterized the process. Moreover, stronger cellular and nuclear alteration as cellular polymorphism, intracellular acidophilic bodies, Mallory bodies, huge nuclei, and nucleoli were evident. The TG livers were also characterized by the presence of inflammation and activated resident immunological cells, perivascular fibrosis, and a variable degree of steatosis (intensity ranged from <5% to 80%) but not advanced fibrosis or cirrhosis. The diagnosis of HCC was confirmed by the absence of reticulum network with Gomori staining (Fig. 1B, Table 1).

**Table 1:** Histological features.

Age (months)	Genotype	n	Steatosis grade	Fibrosis stage	Inflammation score
			G1: G2: G3: G4	F0: F1: F2: F3: F4	0: 1: 2: 3
3	WT	5	5: 0: 0: 0	0: 5: 0: 0: 0	3: 2: 0: 0
	TG	5	5: 0: 0: 0	0: 5: 0: 0: 0	1: 4: 0: 0
6	WT	5	5: 0: 0: 0	2: 3: 0: 0: 0	4: 1: 0: 0
	TG	6	6: 0: 0: 0	0: 6: 0: 0: 0	2: 4: 0: 0
9	WT	3	3: 0: 0: 0	3: 0: 0: 0: 0	1: 2: 0: 0
	TG	3	2: 1: 0: 0	0: 3: 0: 0: 0	2: 1: 0: 0
12	WT	4	3: 1: 0: 0	0: 4: 0: 0: 0	1: 3: 0: 0
	TG	14	3: 10: 1: 0	0: 5: 8: 1: 0	2: 8: 3: 1
≥15	WT	6	6: 0: 0: 0	2: 4: 0: 0: 0	2: 4: 0: 0
	TG	19	3: 6: 8: 2	9: 4: 4: 3: 0	3: 12: 4: 0

Steatosis grade: 0 ≤ 5%, 1 = 5–33%, 2 = 33–66%, 3 ≥ 66%. Fibrosis stage: F0 = none, F1 = mild/moderate fibrosis, F2 = perisinusoidal& portal/periportal fibrosis, F3 = bridging cirrhosis, F4 = cirrhosis. Inflammation score: 0 = no foci; 1 ≤ 2 foci per 200X field; 2 = 2–4 foci per 200X field; 3 ≥ 4 foci per 200X field.

### HBsAg staining

The presence of HBsAg protein on liver tissues was detected by immunostaining. As expected, HBsAg staining observed only in TG livers. At 3 months, most of the hepatocytes were HBsAg+ with different degrees of intensity. Starting from 6 months negative hepatocytes areas appeared and persisted during aging (Fig. 1C).

### Quantification of serum transaminases

Serum alanine aminotransferase (ALT) and serum aspartate aminotransferase (AST) were quantified in 95 TG and 84 WT animals as markers of hepatocyte damage. In WT, ALT were comparable among ages ranging between 19 and 149 IU/L. No statistical difference was observed during aging with the exceptions of 6 *vs* ≥15 months (p < 0.05) and 12 *vs* ≥15 months (p < 0.005). In TG, ALT was highly variable ranging between 31 IU/L and 740 IU/L. Starting from 3 months, ALT level was more than two times higher in TG compared to their relative WT counterpart (p < 0.001 for all matched groups).

In WT, AST ranged between 52 and 327 IU/L and no statistical difference was observed among ages. In TG, serum AST value ranged from 60 to 699 IU/L. Starting from 6 months, AST level were about two times higher in TG compared to their relative WT counterpart (6 and ≥15 months p < 0.01; 9 and 12 months p < 0.001) (Table 2).

**Table 2:** Body weight and serum transaminases.

Parameter		Median (IQR)				
	Age (months)	3	6	9	12	≥15
	n (WT:TG)	13:11	18:16	18:19	20:31	15:18
Weight (g)	WT	28.4 (*3*.*4*)	34.1 (*5*.*9*)	37.9 (3.95)	40.9 (*7*.*87*)	38 (*2*.*5*)
	TG	29 (*7*.*1*)	34.7 (*5*.*1*)	38.0 (5)	38.9 (*7*.*3*)	38.5 (*3*.*82*)
ALT (IU/mL)	WT	41 (*26*)	44 (*21*.*2*)	38 (26)	48.5 (*26*.*5*)	35 (*10*.*5*)
	TG	98 (*29*.*7*)***	147 (*58*)***	155 (97.5)***	151 (*100*.*5*)***	116.5 (*60*.*5*)***
AST (IU/mL)	WT	88 (*35*)	83 (*56*)	109.5 (56)	90.5 (*62*)	90 (*41*.*5*)
	TG	107.5 (*106*)	188 (*80*)**	205 (110.5)***	189 (*114*.*5*)***	139 (*82*.*2*) **

Student’s t-test with Welch correction *p < 0.05, **p < 0.01, ***p < 0.001; WT *vs* TG age matched. IQR = interquartile range.

### Analysis of the expression of SC and CSC markers in TG mouse liver tissue

**Figure 2:**
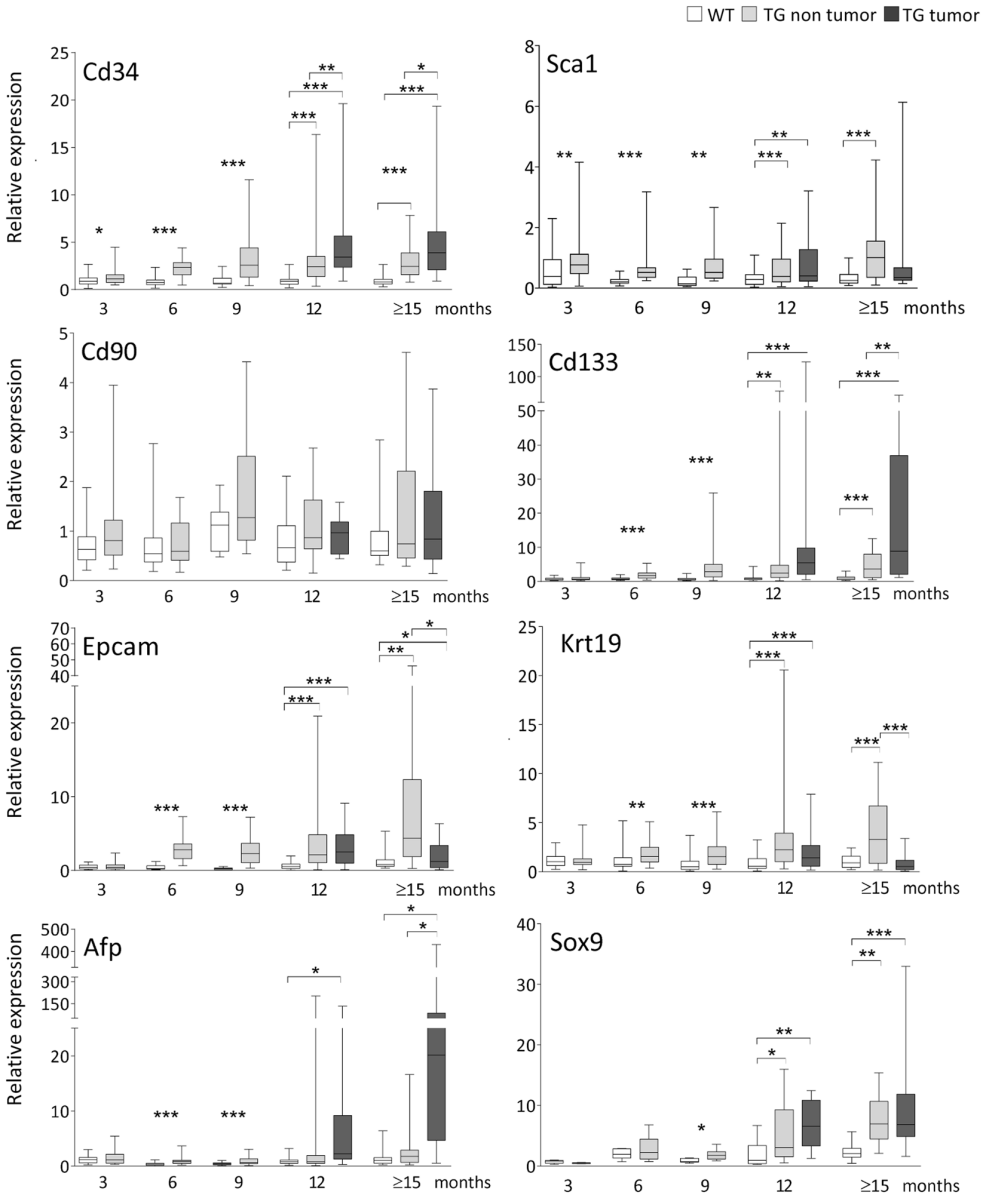
mRNA expression of SC and CSC markers in liver tissue of WT and TG mice. Gene expression is normalized to Gapdh and Bact reference genes and is expressed as AU compared to one WT sample at 3 months defined as 1.00 AU. WT = wild type, TG = transgenic. White box: WT tissue; light grey box: TG non-tumoral tissue; dark gray box: TG tumoral tissue. Student’s t-test with Welch correction *p < 0.05, **p < 0.01, ***p < 0.001.

**Figure 3:**
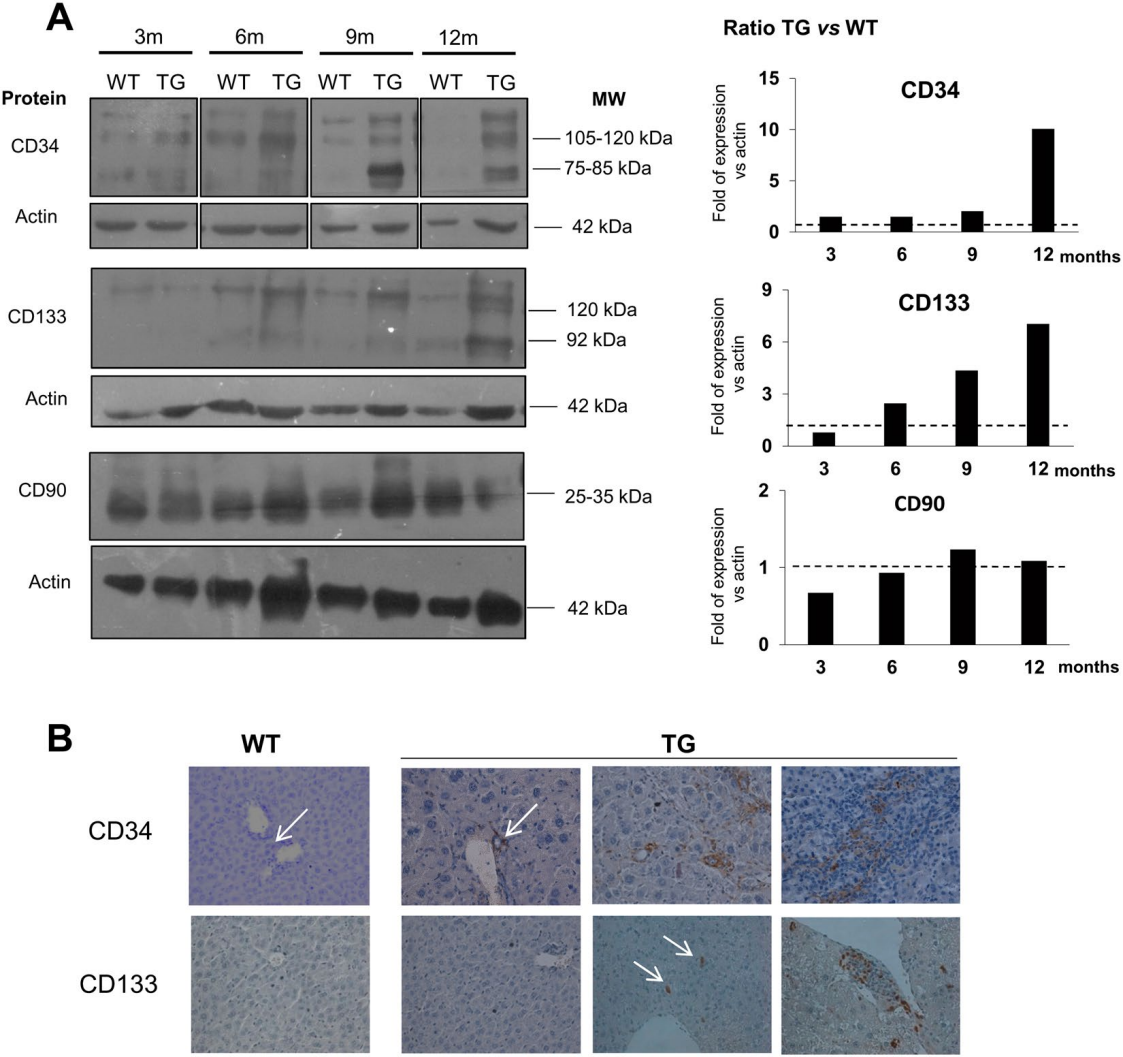
Protein expression in total liver tissue. (**A**) Representative Western blot analysis of CD34, CD133, and CD90 (left panel). Relative protein quantification with bars represent the ratio of TG:WT of two different samples for each genotype (right panel). Actin was used as housekeeping protein. WT = wild type, TG = transgenic. Full-length blots are presented in Supplementary Fig. S1. (**B**) Immunostaining of CD34 and CD133 protein in liver tissue of WT and TG mice. Representative pictures of WT and TG livers during the development of cancer. Objective 40X.

We analyzed the mRNA expression of the normal haematopoietic SC and CSC markers, Cd34, Sca1, Cd90 (Thy1), Cd133 (Prom1), and Epcam, and hepatic progenitor cells Krt19, Afp, and Sox9, at selected age points of 3, 6, 9, 12, and ≥15 months in TG and WT livers. TG liver samples at 12 and ≥15 months were divided in two groups of non-tumoral and tumoral samples based on the macroscopic appearance of the liver tissue. The mRNA level was expressed as arbitrary unit (AU) compared to one WT sample at 3 months defined as 1.0 AU (Fig. 2). Protein expression of CD133, CD34, and CD90 was evaluated by Western Blot analysis (Fig. 3A). The distribution and positivity of CD133 and CD34 cells in liver tissues was conducted by immunohistochemistry on paraffin-embedded tissue sections (Fig. 3B).

#### Expression of haematopoietic SC markers CD34, Sca1, and CD90

The mRNA of Cd34, Sca1, and Cd90 (Thy1) were expressed in all tissues analyzed. As expected, in WT livers, their expression was homogeneous both within the same age group and among the different ages.

In TG livers, the Cd34 mRNA expression showed an increasing pattern with the progression of the disease. Compared to WT, Cd34 expression was significantly up-regulated starting from the first hepatic damage at 3 months and progressively increased during aging. The tumoral TG tissues showed high variability of Cd34 with expression ranging from 0.9 to 19.6 AU at 12 months and from 0.9 to 19.3 AU at ≥15 months. Furthermore, in the tumor, the Cd34 expression was significantly up-regulated compared to both TG non-tumoral (p < 0.01 at 12 months and p < 0.05 at ≥15 months) and WT groups (p < 0.001) (Fig. 2). Protein analysis confirmed this pattern. In WT liver, CD34 protein was stable during aging, while in TG samples an increased level was observed during the progression of the disease (Fig. 3A). Immunostaining of CD34+ cells in WT liver revealed that these cells were confined to the portal triad while in TG liver, the number of CD34+ cells progressively increased and scattered from the perivascular area into the parenchyma. CD34+ cells were small with scant cytoplasm and grouped in clusters (Fig. 3B).

As for Cd34, the Sca1 mRNA expression in TG samples showed a slight but significant up-regulation starting from 3 months (p < 0.01) and was continuously observed in non-tumoral tissue (6, 12 and ≥15 months p < 0.001, 9 months p < 0.01 compared to WT). At 12 months, in tumoral samples, Sca1 was up-regulated compared to WT (p < 0.01) while at ≥15 months, tumoral expression was more variable compared to non-tumoral tissues, even though the median value was lower compared to non-tumoral (1.0 AU compared to tumoral 0.4 AU). No statistical differences were observed among TG groups (Fig. 2).

Cd90 expression was found to be relatively stable during aging and/or hepatocarcinogenesis, and no statistical differences were observed in TG livers compared to WT counterparts at both mRNA and protein level. (Fig. 3A,B).

#### Expression of CSC markers CD133 and Epcam

The mRNA of Cd133 and Epcam were expressed in all tissues analyzed. In WT livers, their expression was homogeneous both within the same age group and among the different ages. On the contrary, in TG livers, the Cd133 expression started to increase at 6 months (p < 0.001 compared to WT) and progressively increased during the progression of the disease compared to matched WT (p < 0.001 at 6, 9, 12 non-tumoral, and ≥15 non-tumoral months, p < 0.01 in 12 months non-tumoral). A significant increase was also observed within the TG groups in accordance to the progression of the disease (3 *vs* 6 months p < 0.001; 6 *vs* 9 and 9 *vs* 12 non-tumoral months p < 0.01). In tumoral samples, the Cd133 expression was very variable ranging from 0.4 to 122.7 AU at 12 months (median value 5.5 AU) and from 1.1 to 71.0 AU at ≥15 months (median value 8.9 AU) (Fig. 2). In accordance with mRNA expression, Prom1 (CD133) protein was very low in WT liver while it was increased in TG samples during the progression of the disease (Fig. 3A). Despite the evidence of Prom1 protein by Western blot, we did not observe Prom1 + cells in WT liver tissue, while in TG they appeared scattered into the parenchyma at 12 and ≥15 months (Fig. 3B).

In line with Cd133 distribution, Epcam mRNA expression in TG samples showed a significant up-regulation at 6, 9, 12 (tumoral and non-tumoral) and 15 (non-tumoral) months compared to WT (p < 0.05 at 15 months, and p < 0.001 for all the other time points). On the contrary, at ≥15 months, tumoral tissue showed a significant decrease compared to non-tumoral (p < 0.05) (Fig. 2).

#### Expression of hepatic progenitor cells markers Krt19, Afp, and Sox9

The mRNA of Krt19, Sox9, and Afp were expressed in all tissues analyzed. In TG livers, starting at 6 months of age, Krt19 mRNA expression progressively increased in the non-tumoral TG samples compared to the age-matched WT. Within the TG groups, a significant increase was observed moving from 3 to ≥15 months (p < 0.05). Similarly to Epcam, Krt19 expression was lower in tumoral tissue compared to non-tumoral at both 12 and ≥15 months (Fig. 2).

The Afp mRNA expression in WT livers was very low. In the TG tissue, Afp started to be up-regulated from 6 months of age (p < 0.001 at 6 and 9 months *vs* WT) with a drastic up-regulation in the tumoral tissue at 12 (p < 0.05 *vs* WT) and ≥15 months (p < 0.05 *vs* WT and TG non-tumoral). (Fig. 2).

The expression of Sox9 mRNA was found to be variable among ages, both in WT and TG livers. In TG samples, we observed a significant increased expression starting from 9 months (p < 0.05 *vs* WT). At 12 and ≥15 months, the median value was 3.0 and 6.9 AU in non-tumoral tissue, and 6.5 and 6.8 AU in tumoral tissue, respectively. Sox9 was significantly up-regulated compared to WT counterpart and compared to 9 months TG (9 TG *vs* 12 TG nontumoral p < 0.05, 9 TG *vs* 12 TG tumoral, p < 0.01, 9 TG *vs* ≥15 months nontumoral and tumoral, p < 0.001) (Fig. 2).

### Differential expressions between paired tumoral and adjacent tissues

**Figure 4:**
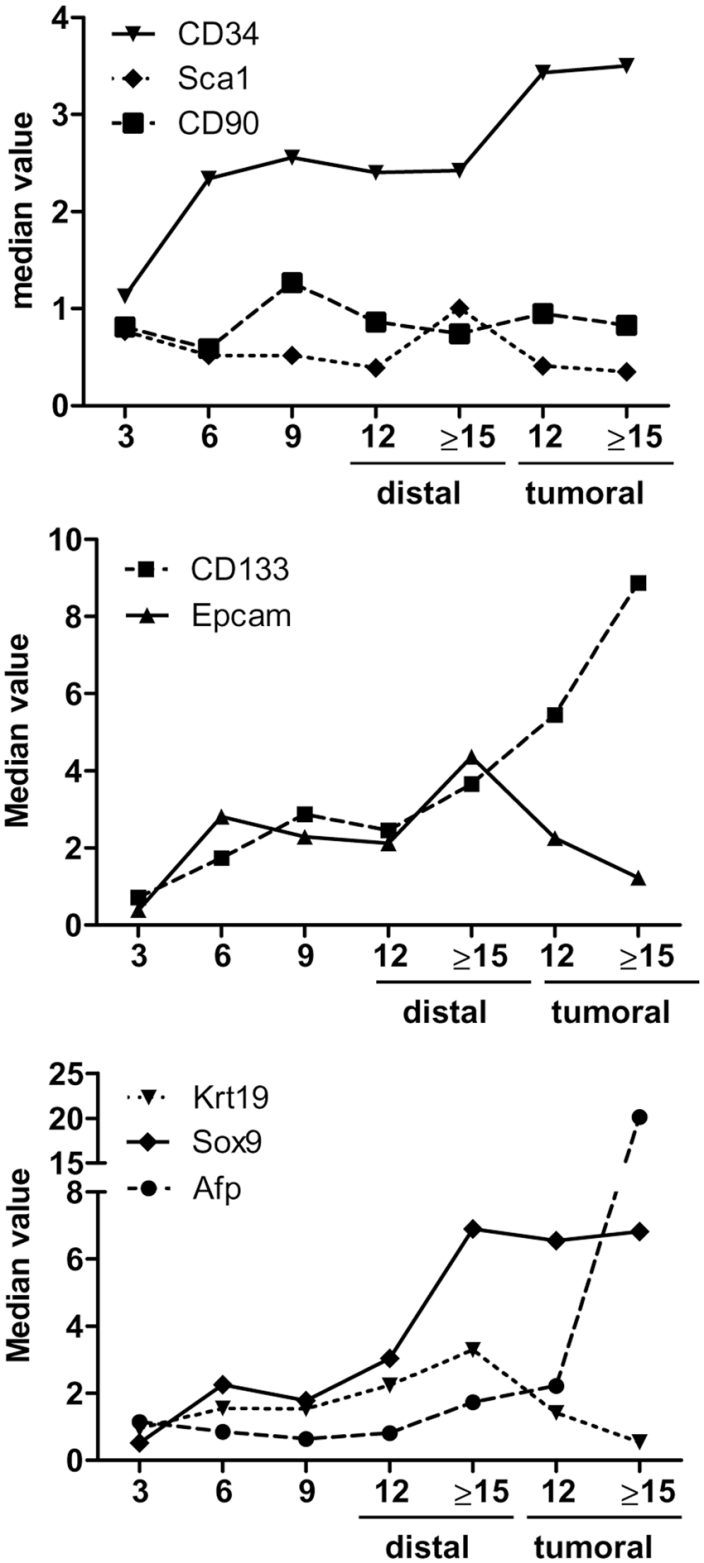
Dynamics of gene expression trend in TG mice liver during hepatocarcinogenesis. (**A**) Hematopoietic SC markers Cd34, Sca1, and Cd90. (**B**) CSC markers Cd133 and Epcam. (**C**) Hepatic progenitors cells markers Krt19, Afp, and Sox9. Values are reported as median.

Along the course of liver damage in TG mice, we observed the dynamics of gene expression of SC markers (Fig. 4). Due to the high variability in gene expression patterns observed in TG tissues, especially in the later phases of the hepatocarcinogenesis, we compared gene signatures in paired tumoral and non-tumoral tissue. We checked 35 paired tissues from 23 TG animals at 12 and ≥15 months. The differential expression was calculated as the ratio of tumoral/non-tumoral mRNA. A ratio value higher than 1.0 indicated folds of higher expression in tumoral tissues.

**Figure 5:**
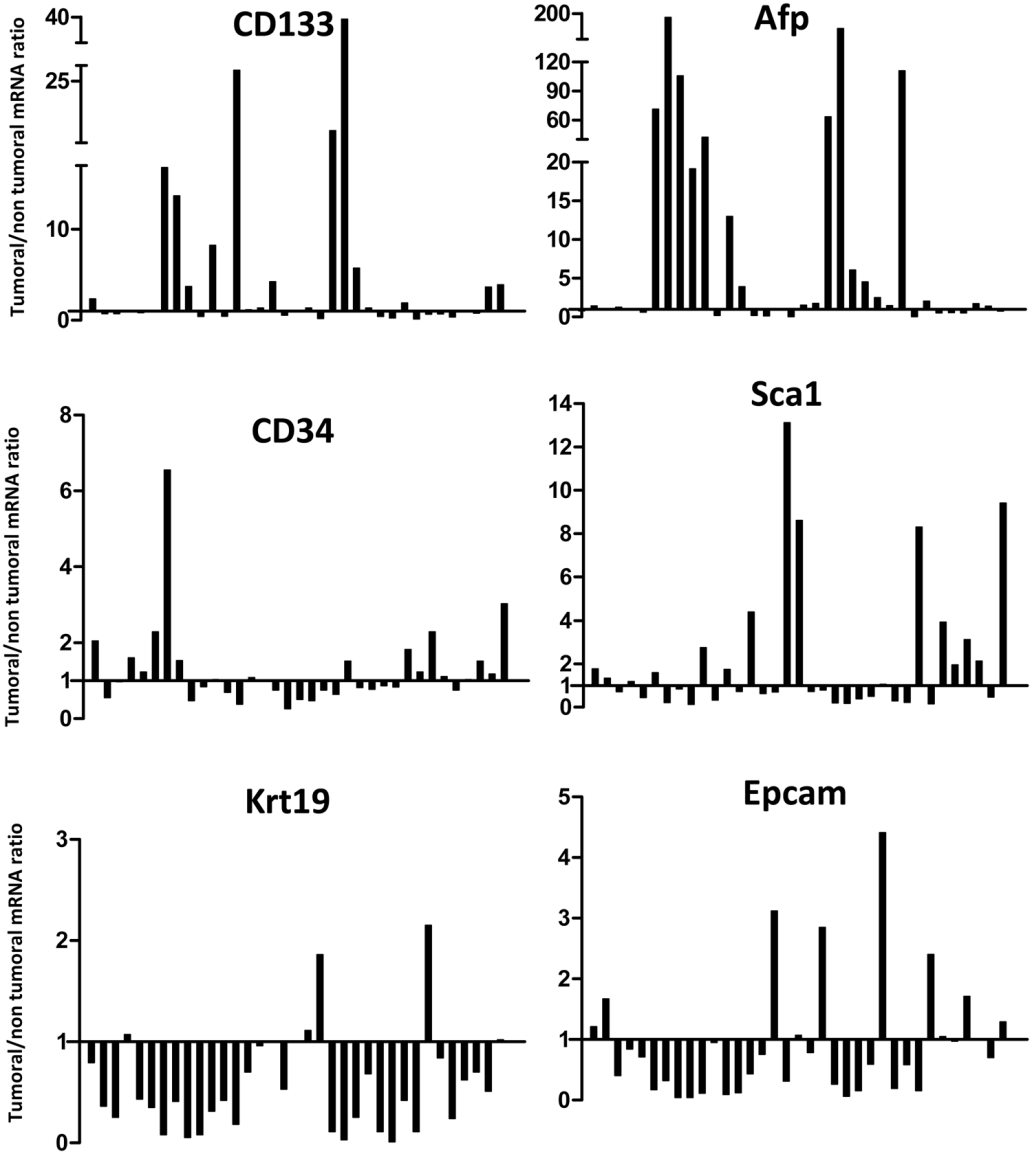
mRNA expression ratio between tumoral and nontumoral tissues within the same TG animal. The ratio between mRNA expressions was evaluated in 39 paired tissues. Value of ratio >1.00 indicated higher expression in tumoral compared to nontumoral.

As shown in Fig. 5, intra-variability within animal was very high. The ratio of Cd34 ranged from 0.3 to 6.6, with a tumoral/non-tumoral ratio >1.2 found in 12 (34%) paired tissues. The ratio of Cd133 ranged between 0.1 and 39.6, with a tumoral/non-tumoral ratio >1.2 found in 16 (46%) paired tissues, with an evident increase >10 folds in 5 samples (14%). The ratio of Afp was variably up-regulated in 21 (60%) paired tissues ranging between 1.3 and 194.0 folds; 9 samples showed a strong up-regulation >10 folds. The tumoral/non-tumoral ratio of Sca1 >1.2 was found in 14 (40%) paired tissues with fold-increase between 1.3 and 13.1.

Opposite pattern was found for Krt19 and Epcam. The Krt19 was down-regulated in tumoral samples in 28 (80%) paired tissues and showed a little up-regulation (between 1.1 and 2.1 folds) in only 4 samples, while Epcam was down-regulated in 27 (77%) of tumoral samples. Of notice, the samples with a marked up-regulation of Cd133 also showed high expression of Afp and down-regulations of Krt19, Epcam, and Sca1.

### Association between CSC markers and liver pathology

To investigate the link between the dysregulation of SC and CSC genes with specific pathologies, we evaluated their expression in 31 TG samples grouped based on the histological diagnosis and reticulum staining in HCC (n = 5), dysplasia (n = 12), and early hepatic alterations (n = 14). Nuclear pleomorphism, cellular edema, and apoptotic bodies mainly characterized early hepatic alterations.

**Figure 6:**
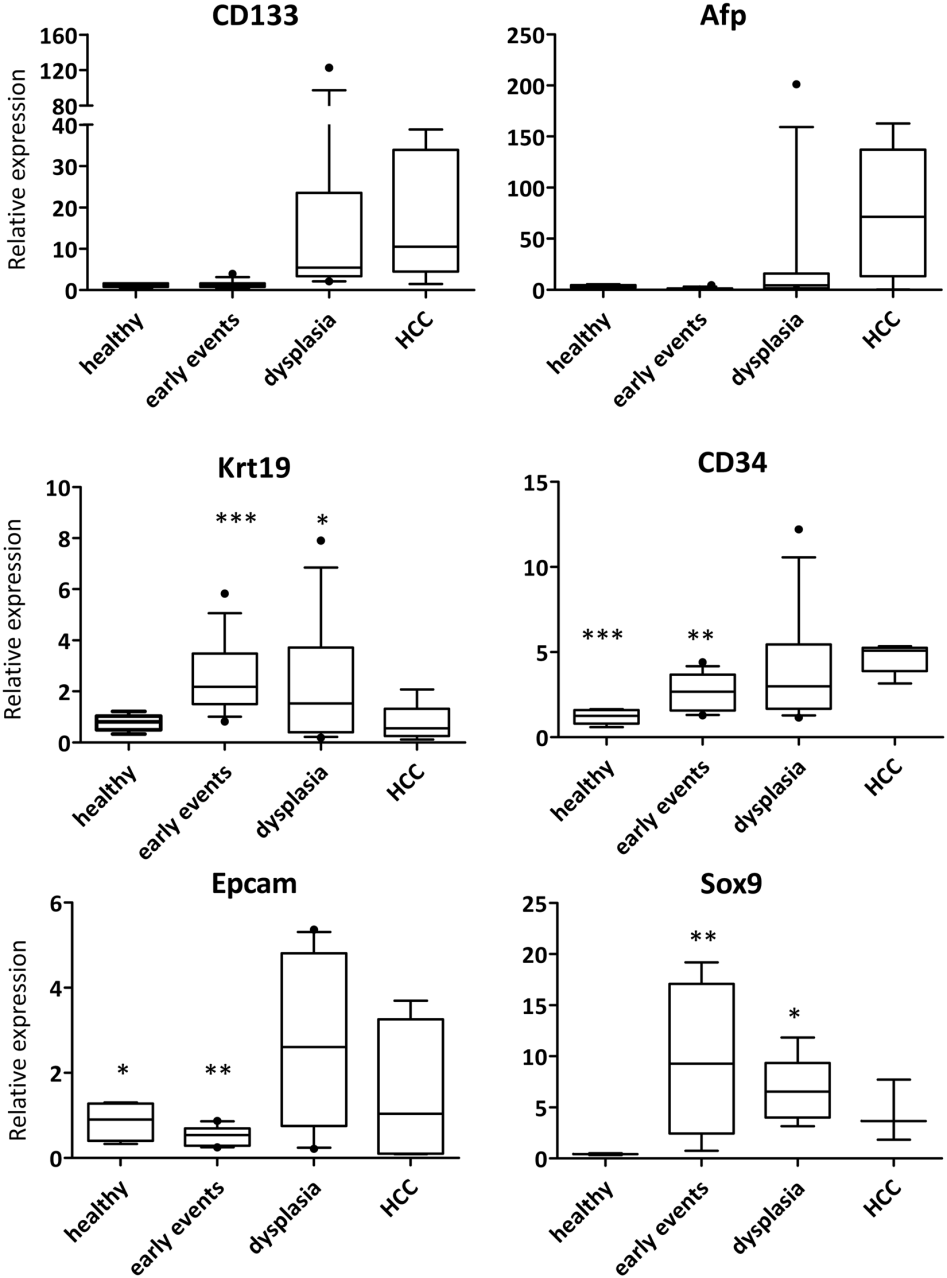
Expression of SC and CSC markers mRNA in TG liver tissue in different pathologies. mRNA expression is normalized on Gapdh and Bact reference genes and is expressed as AU compared one sample WT at 3 months set as 1.00 AU. Total n: 35 TG tissues (HCC [5], dysplasia [12], early events [13], healthy [5]). Cd34: p-value *vs* HCC; Krt19 and Sox9: p value *vs* healthy, Epcam: p value *vs* dysplasia. Student’s t-test with Welch correction *p < 0.05, **p < 0.01, ***p < 0.001.

As shown in Fig. 6, the expression of Cd133, Afp, and Cd34 progressively increased from healthy (WT), early hepatic injury, dysplasia, and HCC. On the contrary, Krt19 expression progressively decreased from early events to HCC while Epcam expressions showed a significant increase from early events to dysplasia and then a progressive decrease in the HCC group.

## Discussion

Increasing evidences highlight the importance of adult hepatic SC in the regenerative process in chronic liver diseases. In these conditions, hepatocyte senescence leads to the activation of the SC that rapidly replicate and differentiate into both hepatocytes and cholangiocytes maintaining the functionality of the affected liver. On the other side, due to their self-renewing properties and differentiation capacity, hepatic SC can play a key role in carcinogenesis.

In the study of HCC mechanism, the CSC, which is responsible for sustaining the tumor, can derive from both the malignant transformation of SC and the dedifferentiation of hepatocytes. A breakthrough paper by the group of Thorgeirsson demonstrated that the hepatic CSC could be induced from different steps of hepatic cellular maturation ranging from the primary hepatic progenitor cells, lineage-committed hepatoblasts to differentiated hepatocytes. Interestingly, the CSC originated from different cell types showed differential gene expression profiles, and induced a specific HCC subtype in accordance with their origin^9^.

In this study, we employed the C57BL/6J-TG(ALB1HBV)44BRI/J transgenic mouse that develop a progressive liver damage from early inflammation to HCC due to the accumulation of the HBsAg protein within the hepatocytes^6,8^. Starting from 3 months, these mice show a dysregulation in genes involved in apoptosis, cell cycle, NF-κB pathway, and inflammatory response^10^.

In particular, we focused our analysis on the evaluation of the liver damage during the life span of the mice (from 3 until 18 months of age) and its correlation with SC and CSC compartment activation during the progression of the disease. Liver damage was assessed by the measurement of ALT and AST together with the histological analysis. High levels of serum ALT and AST in TG mice started at 3 months of age showed a progressive liver damage. Meanwhile, we observed a delay of the histological damage with respect to the referred literature^9^. The activation of SC compartment during the progression of the disease and the appearance of CSC were followed with the expression of the most common hepatic SC and CSC markers: Cd34, Sca1, Cd90 (Thy1), Cd133 (Prom1), Epcam, Krt19, Afp, and Sox9. Their expression was homogeneous and lower in WT tissue compared to TG.

In accordance to what we observed from the histological analysis and serum transaminases levels, a high variability in the expression of SC markers was observed among animals during hepatocarcinogenesis, especially in the more advanced stages (≥12). We found that hematopoietic SC markers Cd34 and Sca1 were up-regulated as early as 3 months during the inflammation phase. Interestingly, starting from 6 months of age (early hepatocyte damage), the CSC markers Cd133 and Epcam, and hepatic SC Krt19, Afp, and Sox9 clearly showed a progressive increase.

This pattern was even clearer when we analyzed the gene expression based on histological finding. The comparison of these markers with different stages of the liver damages (early hepatic alterations, dysplasia, and HCC), independently from the age of the animals, showed an increasing trend for Cd133, Cd34, and Afp from early liver damage to dysplasia and HCC. However, Krt19 and Epcam expressions showed an increasing pattern from inflammation to dysplasia, but their expressions decreased when HCC developed. Krt19 is expressed in a subset of HCC with poor prognosis and it was correlated with increased tumor size, decreased differentiation, epithelial-mesenchymal transition, metastasis, and microvascular invasion^11^. As described by Chisari, our animal model develops tumors that, despite hepatic aggressiveness, do not show metastasis supporting the hypothesis that Krt19+ tumors are more aggressive and more prone to metastasis. Interestingly, the only two tumors with higher Krt19 in tumor compared to non-tumoral also showed high expression of Epcam, down-regulation of Cd133, and a slight up-regulation of Afp.

CD90 (Thy1) showed a modest dysregulation. In the liver, CD90 is expressed by different cells type. In addition to hepatic SC/CSC, fibroblasts and bone marrow derived cells express this marker. In particular, during wound healing and fibrosis-activated myofibroblasts express high level of this marker^12^. Previously, we reported that the CD90 was up-regulated in human liver cirrhosis and HCC compared to normal tissue^13^. In this model, no advanced fibrosis and cirrhosis were observed during hepatocarcinogenesis, corroborating the idea that CD90+ cells in the tumor are not derived directly from hepatic SC.

Combination of different CSC marker had been used to stratify HCC and predict outcome and survival. Different combinations of CSC markers, although sometimes contradictory, were reported to have higher tumorigenicity capacities to its related negative counterpart. In this model, expression of SC and CSC markers showed high variability leading to the future possibility to study different CSC subpopulations. Apart from Afp and Sox9, Cd133 showed the higher up-regulation (40-fold) in HCC and its expression was associated with higher expression of Afp. Conversely, a tumor with higher expression of Epcam showed down-regulation or low up-regulation of Cd133 (within 4 folds) and Afp (within 2.5 folds) compared to its paired tissue.

Several reports described the involvement of HBV in the generation of CSC, especially for the HBx protein. The pluripotent stem cell transcription factors Oct-4, Nanog, and Klf-4, as well as EpCAM and β-catenin, are up-regulated in HBx expressing cells^14^. Moreover, clinical evidence showed that high HBx expression in human HBV-related HCC was statistically associated with the expansion of EpCAM + or OV6 + tumor cells, and aggressive clinicopathological features^15,16^. On the other hand, information of the oncogenicity of HBsAg is still limited. Recently, it had been reported that PreS1 activated the expressions of CSC markers CD133, CD117, and CD90 in normal hepatocytes and HCC cells, indicating the new role of PreS1 in the appearance and self-renewal of CSC during HCC development^17^.

In conclusion, our data showed that early liver inflammation caused by the HBsAg accumulation activates the SC population. The gene expression data may indicate a possible oncogenic mechanism starting by the up-regulation of SC Sca1 and Cd34. The progression of liver damage induces the compartment of hepatic SC Sox1, Krt19, and Afp, together with the appearance of potential CSC Cd133 and Epcam. The progressive increase of CSC markers indicates the importance of HBsAg as an oncogenic factor during hepatocarcinogenesis. Further studies are needed to elucidate different mechanisms on the oncogenicity of HBsAg on different stem cells populations.

## Materials and Methods

### Mouse model: samples collection

Male heterozygous C57BL/6J-TG(ALB1HBV)44BRI/J transgenic mice (TG)^7^ breeders were kindly given by the Department of Clinical and Biological Sciences of the University of Turin, Italy. Female breeders C57BL/6 J (WT) were purchased from Charles River (Charles River Laboratories Italia, SRL, Lecco, Italy). All animals were maintained at the animal facility of the University of Trieste, Italy. Experimentation was carried out in accordance with the Guide for the Care and Use of Laboratory Animals. The protocol and animal study were approved by the ethical committee of the University of Trieste and by the responsible administration of the Ministry of Health of the Republic of Italy (D.M. 57/2012-B). Experimental male heterozygote TG and male WT were maintained until they reached the experimental age points. In detail: 17 TG and 16 WT were sacrificed at 3 months, 20 TG and 23 WT at 6 months, 20 TG and 20 WT at 9 months, 38 TG and 30 WT at 12 months, and 19 TG and 16 WT between 15 and 18 months of age.

### Serum transaminase quantification

Sera samples from all animals were collected during sacrifice. Serum alanine aminotransferase (ALT) and serum aspartate aminotransferase (AST) were quantified on a Roche Cobas Analyzer at the IRCCS Burlo Garofolo Children Hospital (Trieste, Italy). A total of 84 WT and 95 TG mice sera were analyzed.

### Histology and staining

Liver tissues were fixed in formalin and included in paraffin block with the automated Sakura method at the Santa Maria della Misericordia Hospital (Udine, Italy). The fixed slices were subjected to hematoxylin & eosin, Gomori, and HBsAg staining. Histological analysis and diagnosis were performed by a single pathologist for a minimum of three different liver sections, both for each age and strain.

### Western blot

Liver tissue was ice-cold homogenized in tissue lysis buffer. Protein extract was size-separated and electro-transferred onto immuno-blot PVDF membrane. The membranes were incubated overnight at 4°C with appropriate antibody based on the manufacturers’ protocol. Protein bands were visualized with the ECL-Plus Western blot detection system solutions (ECL Plus Western blotting Detection Reagents, GE-Healthcare Bio-Sciences, Italy). Primary antibodies used were anti-CD34 (clone MEC14.7, Santa Cruz Biotechnology, Dallas, USA), anti-CD133 (clone 13A4, eBioscience, Milan, Italy), anti-CD90 (clone OX7, Santa Cruz Biotechnology), anti-actin (clone A2066, Sigma-Aldrich).

### Immunohistochemistry

Paraffinated hepatic slices were de-paraffinization with xylene and rehydrated with a gradual concentration of ethanol. Tissues were incubated with the appropriate primary antibody for overnight at 4°C based on the manufacturers’ protocol, followed by incubation with the secondary anti-rat-biotinylated antibody (Vectastain Elite ABC Kit). The complex streptavidin-biotin was visualized with the DBA peroxidase substrate kit (Vector Laboratories, UK). Slides were observed under a Leica DM2000 microscope (Leica Camera AG, Solms, Germany). Primary antibodies used were anti-CD34 (clone MEC 14.7, Santa Cruz Biotechnology, Dallas, USA) and anti-CD133 (clone 13A4, eBioscience, Milan, Italy).

### Gene expression analysis

Total RNA from liver tissue was extracted using the EuroGold RNA Pure (EuroClone, Milan, Italy) and reversed transcribed using an iScript cDNA synthesis Kit (Bio-Rad Laboratories, Hercules, CA, USA) based on the manufacturers’ protocol. The qRT-PCR was performed according to the SYBR Green Supermix (Bio-Rad) protocol. Reactions were run on the IQ5 PCR detection system (Bio-Rad). The mRNA relative expressions were calculated by using IQ5 software version 3.1 (Bio-Rad). The primers for qRT-PCR were designed using software Beacon Designer Version 7.9 (Premier Biosoft International, Palo Alto, CA, USA).

The sequences of primers used were as follows: B-actin (5′-CCTTCTTGGGTATGGAATCCTGTG-3′; 5′-CAGCACTGTGTTGGCATAGAGG-3′), Gapdh (5′-CCAGTATGACTCCACTCACG-3′; 5-’CTCGCTCCTGGAAGATGGTG-3′), Cd133 (5′-GACATCTCAGTTGATTCCAAGG-3′; 5′-CATGGCGCATTCTGCTTCTGC-3′), Cd34 (5′-ATATGCTTACACATCATCTTCT-3′; 5′-AACCTCACTTCTCGGATT-3′), Krt19 (5′-CAGGTCAGTGTGGAGGTG-3′; 5′-TCAATCCGAGCAAGGTAGG-3′), Afp (5′-GTTCCTTATTGGTTACACGAG-3′; 5′-CAGGGCTTGCTTCATTCC-3′), Cd90 (5′-AACTTCACCACCAAGGAT-3′; 5′-TTGTCTCTATACACACTGATACT-3′), Epcam (5′-ATTGTGGTGGTGTCATTAG-3′; 5′-TCCTTTATCTCAGCCTTCT-3′), Sca1 (5′-AGGAGGCAGCAGTTATTGTG-3′; 5′-TATTAGGAGGGCAGATGGGTAA-3′), Sox9 (5′-CCTCACTACAGCCCCTCCTA-3′, 5′5′-TCTGATGGTCAGCGTAGTCG-3′).

### Statistical analysis

Box and bar plot graphics and statistical analysis were constructed using software GraphPad Prism Version 5.0 (GraphPad Software, Inc., La Jolla, CA, USA). The student’s t-test (normal Gaussian distribution) was performed for statistical comparison between groups. Value of p < 0.05 was regarded as statistically significant.

## Electronic supplementary material

supplementary data

## Data Availability

The data and images generated during the current study are available from the corresponding author on reasonable request.
