# Supplementary material for: Activation of hepatic stem cells compartment during hepatocarcinogenesis in a HBsAg HBV-transgenic mouse model
Source: Sci Rep. 2018 Sep 3;8:13168. doi: 10.1038/s41598-018-31406-5 (PMC6120871; doi:10.1038/s41598-018-31406-5)
Supplement: Supplementary file 1 — supplementary data [file 41598_2018_31406_MOESM1_ESM.docx]

**Activation of hepatic stem cells compartment during hepatocarcinogenesis in a HBsAg HBV-transgenic mouse model**

Beatrice Anfuso^1^, Korri E El-Khobar^2^, Susan I Ie^2^, Claudio Avellini^3^, Oriano Radillo^4^, Alan Raseni^4^, Claudio Tiribelli^1^, Caecilia HC Sukowati^1*^

1.Fondazione Italiana Fegato, AREA Science Park Basovizza, SS14 km 163.5, 34149 Trieste, Italy

2. Laboratory of Hepatitis and Emerging Diseases, Eijkman Institute for Molecular Biology, Jl. Diponegoro 69, 10430 Jakarta, Indonesia

3. Department of Medical and Biological Sciences, University Hospital, Piazzale Santa Maria della Misericordia 15, 33100 Udine, Italy

4. Laboratory of Clinical Analysis, Children Hospital Burlo Garofolo IRCCS, Viadell'Istria, 65, 34137 Trieste, Italy

*Correspondence and requests for materials should be addressed to CHCS caecilia.sukowati@fegato.it

**Supplementary figure s1.** Unprocessed original scans of Western Blot in figure 3. Analysis of CD34, CD133, and CD90. Actin was used as housekeeping protein. WT=wild type, TG=transgenic.

**
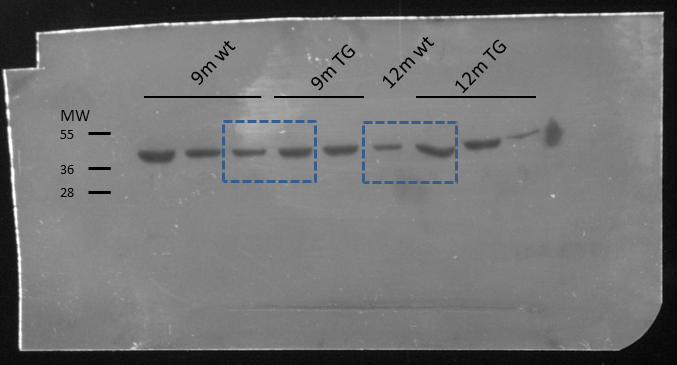
**

**
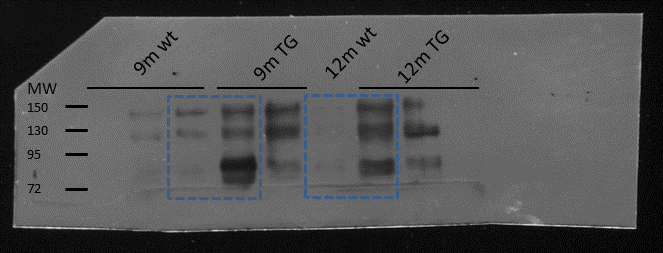

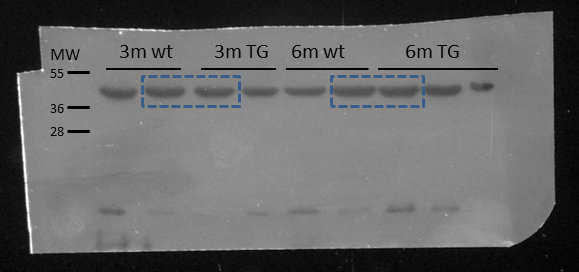

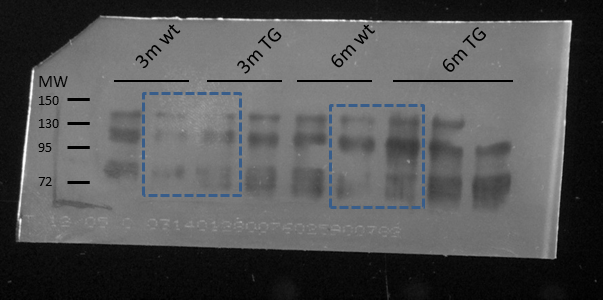
**

**CD34 Actin**

**
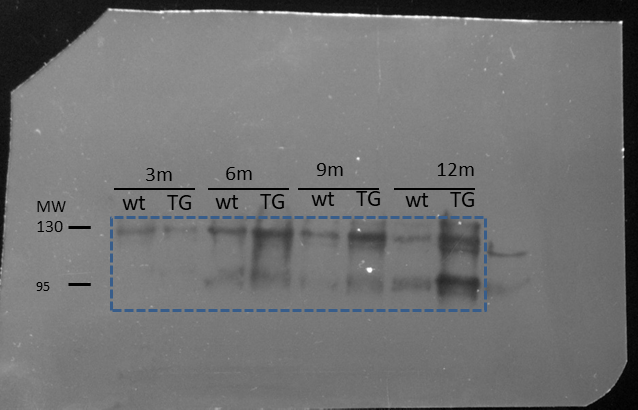

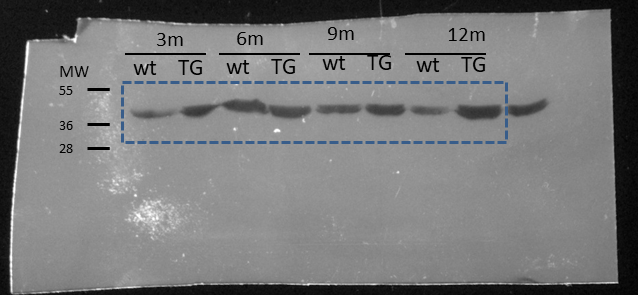
**

**CD133 Actin**

**
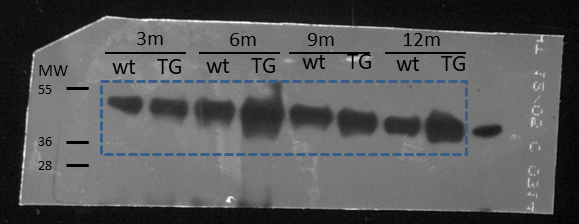

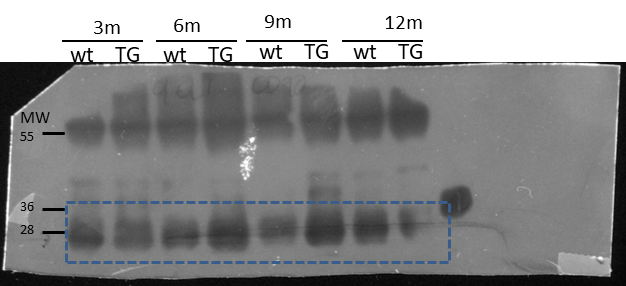
**

**CD90 Actin**
